# Levels and trends of maternal death in Baoan district, Shenzhen, China, 1999–2022

**DOI:** 10.3389/fpubh.2023.1051717

**Published:** 2023-04-17

**Authors:** Wei Wang, Yuanfang Zhu, Yuli Cheng, Xu Chen, Yali Luo

**Affiliations:** ^1^Department of Health Care, Shenzhen Baoan Women's and Children's Hospital, Shenzhen Guangdong, China; ^2^Office of Hospital Director, Shenzhen Baoan Women's and Children's Hospital, Shenzhen Guangdong, China; ^3^Office of Hospital Director, Shenzhen Baoan People's Hospital, Shenzhen Guangdong, China

**Keywords:** maternal death, migrant population, advanced maternal age, levels, trends

## Abstract

**Background:**

China had achieved impressive success in improving maternal health, while the progress of reducing maternal mortality ratio (MMR) varied across regions. Some studies had reported maternal mortality from national or provincial perspective, but researches of the MMR on long-term period at the city or county level rare been reported. Shenzhen has experienced significant socioeconomic and health changes, reflecting the typical development of China's coastal city. This study mainly introduced the levels and trends of maternal death in Baoan district, Shenzhen from 1999 to 2022.

**Methods:**

Maternal mortality data were extracted from registration forms and the Shenzhen Maternal and Child Health Management System. Linear-by-Linear Association tests were used to evaluate the trends of MMR among different groups. The study periods were divided into three stages by 8-year interval and χ^2^ test or *Fisher's* test was used to test the difference in maternal deaths of different periods.

**Results:**

During 1999–2022, a total of 137 maternal deaths occurred in Baoan, the overall MMR was 15.91 per 100,000 live births, declined by 89.31% with an annualized rate of 9.26%. The MMR declined by 68.15% in migrant population, with an annualized rate of 5.07%, faster than that in permanent population (48.73%, 2.86%). The MMR due to direct and indirect obstetric causes shown a downward trend (*P*<0.001) and the gap between them narrowed to 14.29% during 2015–2022. The major causes of maternal deaths were obstetric hemorrhage (4.41 per 100,000 live births), amniotic fluid embolism (3.37 per 100,000 live births), medical complications (2.44 per 100,000 live births) and pregnancy-induced hypertension (1.97 per 100,000 live births), the MMR due to the above causes all shown decreasing trends (*P* < 0.01), pregnancy-induced hypertension became the leading cause of deaths during 2015–2022. The constituent ratio of maternal deaths with advanced age significantly increased by 57.78% in 2015–2022 compared with in 1999–2006.

**Conclusions:**

Baoan district had made encouraging progress in improving maternal survival, especially in migrant population. To further reduce the MMR, strengthening professional training to improve the capacity of obstetricians and physicians, increasing the awareness and ability of self-help health care among elderly pregnant women were in urgent need.

## 1. Introduction

Maternal death remains a major health challenge worldwide. Maternal mortality ratio (MMR) is an important indicator that reflects not only the maternal and child safety, but also the economic, cultural and healthcare level of a country or region (1, 2). The global MMR declined from 281.5 per 100,000 live births in 1990 to 195.7 per 100,000 live births in 2015, with an annual decline of 1.5% (3). Globally, low- and middle-income countries contributed 94% of all maternal deaths occur, most of which could have been prevented (4, 5).

As one of the handful countries that have achieved the Millennium Development Goals 5 (MDG5, namely: 75% reduction of the MMR in 2015 compared with 1990) (6). the Chinese government has implemented multiple national programs to reduce the maternal deaths over the past three decades, such as (i) operating a four-level maternal health services network since the early 1990s, (ii) implementing the “Reducing Maternal Mortality and Eliminating Neonatal Tetanus” project and “In-hospital delivery subsidy for Rural Women” project since 2000 and 2009, respectively (iii) constantly improving maternal healthcare resources and services. Therefore remarkable progress had been made in improving maternal health in China, the national MMR declined from 111.0 per 100,000 live births in 1990 to 21.8 per 100,000 live births in 2015 (6), and to 16.5 per 100,000 live births in 2021 (7).

The progress of lowering MMR varied across regions in China because of socioeconomic imbalance and demographic differences (8, 9). Liang and colleagues (10) pointed out that there existed substantial heterogeneity in MMR at the county level, analyses of levels and trends in MMR at the local level would be helpful to highlight successful experiences and determine what needed to be improved. However, to the best of our knowledge, although previous studies related to maternal mortality had been published from the national or provincial perspective (10–14) with larger population based, researches of the MMR on long-term period at the city or county level had rare been reported in recent years.

Shenzhen city is located in Guangdong Province in the south coast of China (113°43'−114°38'E and 22°24'−22°52' N). As the first Special Economic Zone established by China's reform and opening-up, Shenzhen has undergone rapid urbanization, which enabled it to be one of the most developed first-tier cities in China. Millions of migrants of reproductive age from all over the nation had been attracted to this city since the 1980s (15). According to the latest National Census in 2020, the total population of Shenzhen was 17.67 million, among which 68.5% were migrants. The population of Shenzhen has experienced significant socioeconomic and health changes, reflecting the typical development of China's coastal city. Thus, Shenzhen is an excellent representation for exploring the health levels and changes of China's developed regions.

In light of the above discussion, we select Shenzhen as study site, aiming to use all available data sources to analyze the levels, trends of the maternal deaths from 1999 to 2022. Meanwhile, we also pointed out some new challenges in reducing maternal deaths under the background of the adjustment of fertility policies (16), and proposed the corresponding suggestions, hoping that it can serve as a reference for other developed regions to reduce MMR.

## 2. Methods

### 2.1. Study design and setting

This was a retrospective study conducted in Shenzhen, China between January 1, 1999 and December 31, 2022.

Baoan is located in the northwest of Shenzhen, it is the largest area (387.96 km^2^) and most populous (5.37 million at the end of 2021) among the ten administrative districts of Shenzhen, Since 2000, Baoan has experienced rapid growth in live births (from around 10,000 in 2,000 to nearly 40,000 in 2022) because of the influx of migrant workers. As previously reported (17), the migrants in Baoan mainly worked in traditional manufacturing factories and were characterized by low educational levels, poor socioeconomic status, and unwillingness to seek healthcare knowledge and service, making it challenging to the prevention of maternal death. Therefore, in terms of reducing maternal mortality, the measures taken in Baoan can provide reference for other regions with similar demographic characteristics.

### 2.2. Data sources

Data (maternal death cases, live births, health-service-related indicators, etc.,) from 1999 to 2002 were collected manually from original registration forms (including death/ birth registration cards and annual reports) because the regional electronic database had not been established before 2003 in Shenzhen. Data from 2003 and beyond were derived from Shenzhen Maternal and Child Health Management System (SZMCHS), which is a city-wide, unified, and continuously updated electronic system built based on the Shenzhen Maternal and Child Health Handbook.

All institutions that provide maternal and child healthcare (MCH) services in Shenzhen are using this system to report medical records for both mother and newborn online, such as prenatal examinations, delivery, deaths due to maternal causes, deaths in children younger than 5 years, and birth certificates. As previously described (18), the system has the advantage of high validity and reliability of data utilization because the data were aggregated on a case-by-case basis, which allowing for accurate calculations of MMR and other relevant key variables.

### 2.3. Definitions

According to the WHO, maternal deaths refers to the annual number of deaths from any cause related to or aggravated by pregnancy or its management (excluding accidental or incidental causes) during pregnancy and childbirth or within 42 days of termination of pregnancy, irrespective of the duration and site of the pregnancy (19).

MMR was defined as the number of maternal deaths during a given time period per 100,000 live births during the same time period.

According to the Chinese Maternal and Child Health Monitoring Work Manual (2013 Version), maternal systematic management refers to meeting all the following requirements from pregnancy to 4 weeks after delivery: (i) had the first prenatal examination before 13 weeks of gestation, (ii) had at least five prenatal examinations, (iii) in-hospital delivery, and (iv) had two postnatal visits within 4 weeks after delivery.

Household registry: according to the Chinese current demographic system and Shenzhen demographic monitoring methods (20), the household registry in our study was classified into two types: (i) the permanent population for those who achieved permanent residence certificate or those who without a permanent residence certificate but lived in Shenzhen for more than 1 year, and (ii) the migrant population for those who without a permanent residence certificate and lived in Shenzhen for <1 year.

Results from the maternal mortality review committee: (i) preventable death-refers to the death that can be avoided according to the conditions and technical level of the medical and health facilities and the physical and mental conditions of the pregnant women, but caused by improper handling or mistakes in a certain link, and (ii) unpreventable death-refers to the death that cannot be avoided due to the limitation of the local medical and health care technology.

Classification of causes of death: (i) direct obstetric death -refers to the death caused by obstetric complications during pregnancy, childbirth and puerperium due to intervention, negligence, improper treatment or a series of events caused by these reasons, such as obstetric hemorrhage, amniotic fluid embolism, and (ii) indirect obstetric death- refers to death caused by original diseases or diseases occurring during pregnancy. This disease is not directly caused by obstetric reasons, but can be aggravated and lead to death due to physiological changes during pregnancy. For example, cardiovascular disease, respiratory disease, digestive system disease and so on.

### 2.4. Quality control

**Figure 1:**
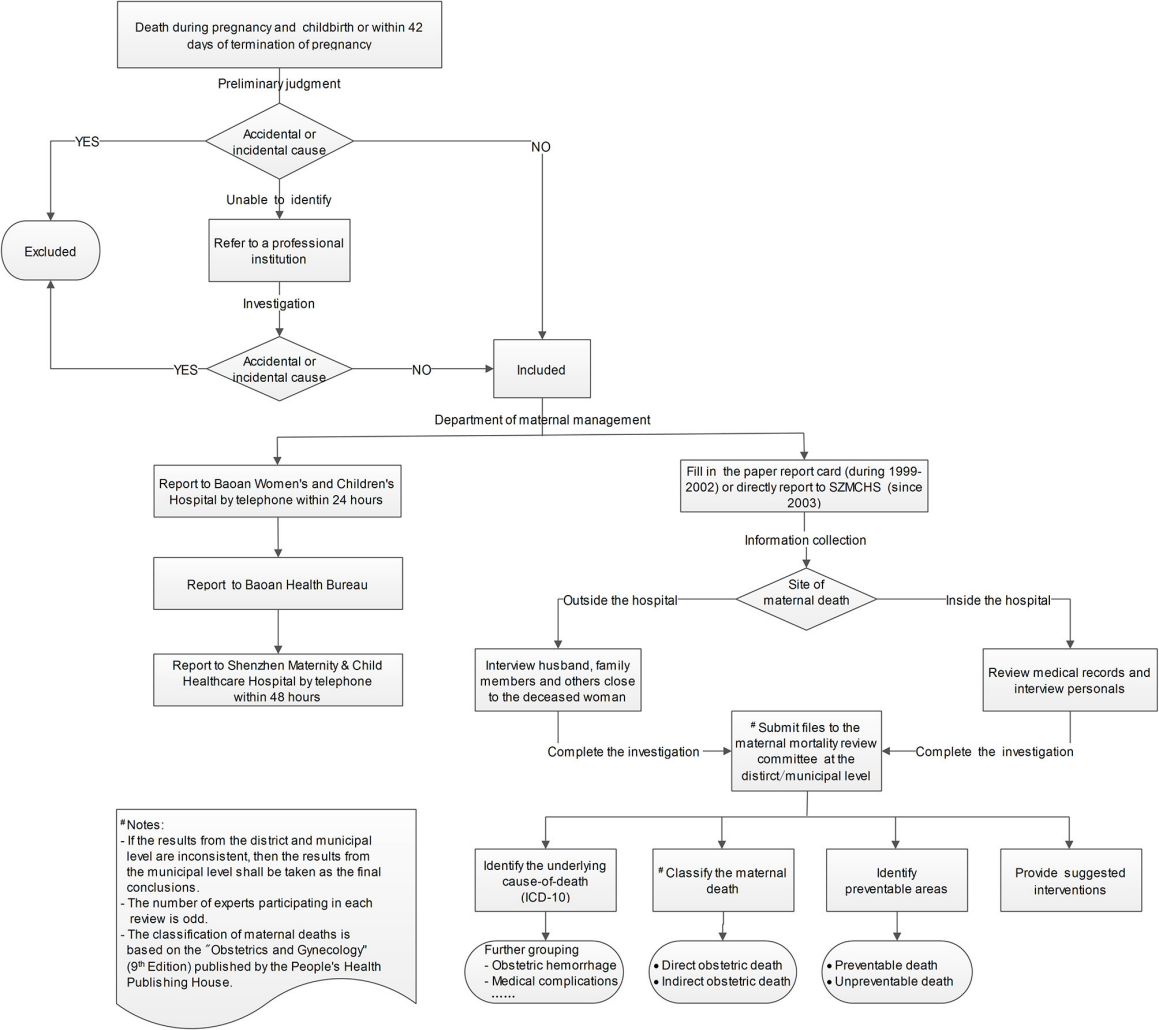
The flow chart of maternal death report and determination of death cause in Baoan district.

The reporting process of maternal death is followed by strict procedures (Figure 1): once pregnancy-related death occurred, department of maternal management should make a preliminary judgment on the death based on the definition of maternal deaths. Since accidental or incidental causes (intentional self-harm, transportation accident, accidental poisoning/overdose, assault/homicide or others) should be excluded, the reporting staffs had been well trained to identify whether it was maternal death. If it could not be accurately identified at the first time, the death case would be referred to a professional institution (such as police station) for investigation. Once a maternal death was confirmed, the relevant department was required to report the death to Baoan Women's and Children's Hospital by telephone within 24 h, and to fill in a report card (during 1999–2002) or immediately input and upload the medical records or other relevant information to the corresponding module of SZMCHS (since 2003).

The SZMCHS also has rigorous quality control mechanisms, including data audits, regular supervision, and standardization of data collection methods. In Shenzhen, every MCH institution had one or two quality-control staffs responsible for routinely checking the authenticity and accuracy of data in the SZMCHS.

The final determination of causes of death also followed by strict and unified criteria (Figure 1): for deaths inside the hospital, information was mainly collected by reviewing medical records and personal interviewing. For deaths outside the hospital, information was collected by interviewing husband, family members and others close to the deceased woman. After completing the investigations of each maternal death, all related files, including investigation form, medical examiner reports, medical records or autopsy reports, were submitted to the maternal mortality review committee at the district/municipal level (established from 2000). The number of experts participating in each review is odd, and unsettled disagreements were resolved by discussions, if a consensus could not be reached, final agreements were reached by the majority of the votes. The underlying cause-of-death, which referred to the disease or injury which initiated the train of events leading directly to death, or the circumstances of the accident or violence which produced the fatal injury, were encoded in accordance with the WHO International Classification of Diseases 10th Edition (ICD-10). After coding, the underlying cause-of-death was further classified into corresponding group (e.g., obstetric hemorrhage,) according to the Chinese Maternal and Child Health Monitoring Work Manual (2013 Version).

### 2.5. Data collection

Information on every maternal death was retrospectively reviewed and extracted independently by two authors (Wang and Zhu): general demographic characteristics (e.g., age, educational level, and household registry), death-related information (e.g., the time and the site of death), and the results from the maternal mortality review committee. Data for all live births during the same period (1999–2022) were also extracted to calculate the MMR. Once data collection was completed, the outliers and missing values were rechecked and supplemented by looking through the original medical records.

### 2.6. Statistical analysis

We firstly calculated the annual MMR in overall, permanent and migrant population respectively, then divided the study period into three stages by 8-year interval to calculate the MMR due to different deaths causes. Linear-by-Linear Association tests were used to evaluate the trend of MMR among different populations and different deaths causes. We also calculated the constituent ratio of certain characteristics (e.g., site of delivery, maternal age) of maternal deaths during different periods. *Pearson*χ^2^ test or *Fisher's* exact test was used to test the difference in maternal deaths of different periods. Epidata 3.0 was applied to set up the database. All statistical analyses were carried out by SPSS, version 21.0 (Chicago, IL, USA). A two-tailed *P*-value of <0.05 was considered statistically significant.

## 3. Results

### 3.1. Trends of the MMR in overall population

During 1999–2022, a total of 137 maternal deaths occurred, and 861,013 live births were born in Baoan district, yielding an overall MMR of 15.91 (95% CI 13.25–18.58) per 100,000 live births. The annual MMR fluctuated from a maximum of 123.39 (95% CI 58.79–188.00) per 100,000 live births in 2,000 to a minimum of zero in 2018 and 2019. The Overall MMR decreased by 89.31%, with an annual decrease of 9.26% ($\chi_{trend}^{2}$ = 156.417, *P* < 0.001) (Table 1).

**Table 1:** The MMR in Baoan district, Shenzhen during 1999–2022.

**Year**	**Permanent population**			**Migrant population**			**Total population**		
	**Live births (n)**	**Maternal deaths (n)**	**MMR (/100,000)**	**Live births (n)**	**Maternal deaths (n)**	**MMR (/100,000)**	**Live births (n)**	**Maternal deaths (n)**	**MMR (/100,000)**
1999	4,813	1	20.78	4,630	8	172.79	9,443	9	95.31
2000	5,098	1	19.62	6,248	13	208.07	11,346	14	123.39
2001	4,503	2	44.41	7,025	8	113.88	11,528	10	86.75
2002	5,555	1	18.00	8,485	12	141.43	14,040	13	92.59
2003	5,152	0	0.00	11,436	6	52.47	16,588	6	36.17
2004	5,641	0	0.00	14,641	7	47.81	20,282	7	34.51
2005	5,937	1	16.84	18,179	7	38.51	24,116	8	33.17
2006	5,806	1	17.22	23,368	3	12.84	29,174	4	13.71
2007	6,818	1	14.67	28,271	8	28.30	35,089	9	25.65
2008	8,580	3	34.97	30,647	9	29.37	39,227	12	30.59
2009	9,626	3	31.17	29,012	6	20.68	38,638	9	23.29
2010	16,960	0	0.00	25,056	5	19.96	42,016	5	11.90
2011	24,891	2	8.04	21,800	1	4.59	46,691	3	6.43
2012	32,525	2	6.15	22,515	1	4.44	55,040	3	5.45
2013	35,474	4	11.28	11,439	1	8.74	46,913	5	10.66
2014	39,155	4	10.22	10,931	1	9.15	50,086	5	9.98
2015	35,013	2	5.71	10,843	1	9.22	45,856	3	6.54
2016	40,181	3	7.47	11,127	0	0.00	51,308	3	5.85
2017	43,540	1	2.30	9,234	1	10.83	52,774	2	3.79
2018	43,463	0	0.00	5,503	0	0.00	48,966	0	0.00
2019	46,289	0	0.00	4,032	0	0.00	50,321	0	0.00
2020	39,608	2	5.05	2,322	0	0.00	41,930	2	4.77
2021	38,572	0	0.00	1,817	1	55.04	40,389	1	2.48
2022	37,547	4	10.65	1,705	0	0.00	39,252	4	10.19
Total	540,747	38	7.03	320,266	99	30.91	861,013	137	15.91
Overall decline (%)			−48.73			−68.15^#^			−89.31
Annual decline (%)			−2.86			−5.07^#^			−9.26
*χ^2^_trend_*			14.900			86.640			156.417
*P*			<0.01			<0.01			<0.01

^#^Since the MMR of migrant population in 2022 was zero, these two ratios were calculated using MMR in 2021 as the data of reporting period.

**Figure 2:**
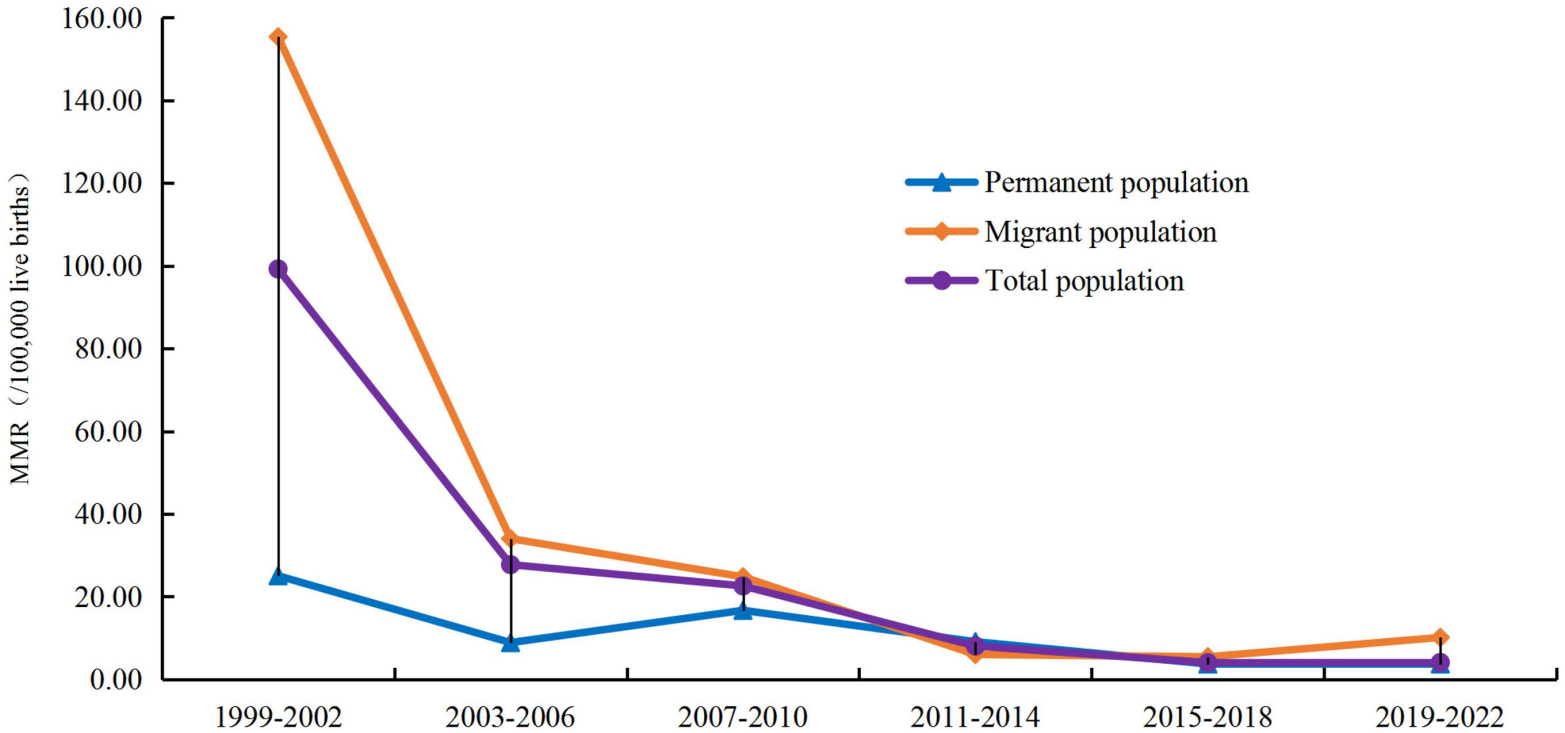
Trends of the MMR in Baoan district during 1999–2022 (using a 4-year interval).

Figure 2 shown the downward trend in MMR when analyzed in 4-year interval, with 4.07 (95% CI 1.06–7.09) per 100,000 live births during 2019–2022 being 95.90% lower than 99.23 (95% CI 70.57–127.89) per 100,000 live births during 1999–2002, which was a significant difference (χ2_*Pearson*_ = 136.172, *P* < 0.001).

### 3.2. Trends of the MMR between permanent and migrant population

Between 1999 and 2022, the majority of maternal deaths occurred in migrant population (99 cases, 72.26%). The MMR in migrant population was 30.91 per 100,000 live births (95% CI 24.82–37.00), which was higher than that in permanent population (7.03 per l00,000 live births, 95% CI 4.79–9.26), with statistically difference (*χ^2^_trend_* = 72.125, *P* < 0.001). MMR in both groups shown a downward trend (Linear-by-Linear Association tests for MMR in both groups *P* < 0.01). The MMR declined by 68.15% in migrant population, with an annual decline of 5.07%, faster than in the permanent population (48.73, 2.86%) (Table 1).

During 1999–2002, the MMR in migrant population was 520.49% higher than that in permanent population (155.37 per 100,000 live births vs. 25.04 per 100,000 per live births, *χ^2^_trend_* = 19.475, *P* < 0.001). But the gap of the MMR between migrant and permanent population rapidly narrowed since 2003 (Figure 2).

### 3.3. Trends of the MMR due to direct and indirect obstetric causes

From 1999 to 2022, there were 90 (65.69%) and 47 (34.31%) pregnant women died of direct and indirect obstetric causes, the corresponding MMR was 10.45 (95% CI 8.29–12.61) per 100,000 live births and 5.46 (95% CI 3.90–7.02) per 100,000 live births, respectively. Both groups shown significant decrease in MMR when analyzed the trend among different research periods (Table 2).

**Table 2:** The MMR due to direct and indirect obstetric causes in Baoan district during different period.

**Period**	**Direct obstetric death**		**Indirect obstetric death**	
	**Maternal deaths (n)**	**MMR (95% CI)**	**Maternal deaths (n)**	**MMR (95% CI)**
1999–2006	48	35.16 (25.22–45.11)	23	16.85 (9.96–23.73)
2007–2014	34	9.61 (6.38–12.84)	17	4.81 (2.52–7.09)
2015–2022	8	2.16 (0.66–3.65)	7	1.89 (0.49–3.29)
Total	90	10.45 (8.29–12.61)	47	5.46 (3.90–7.02)
*χ^2^_trend_*		89.706		34.231
*P*		<0.01		<0.01

**Figure 3:**
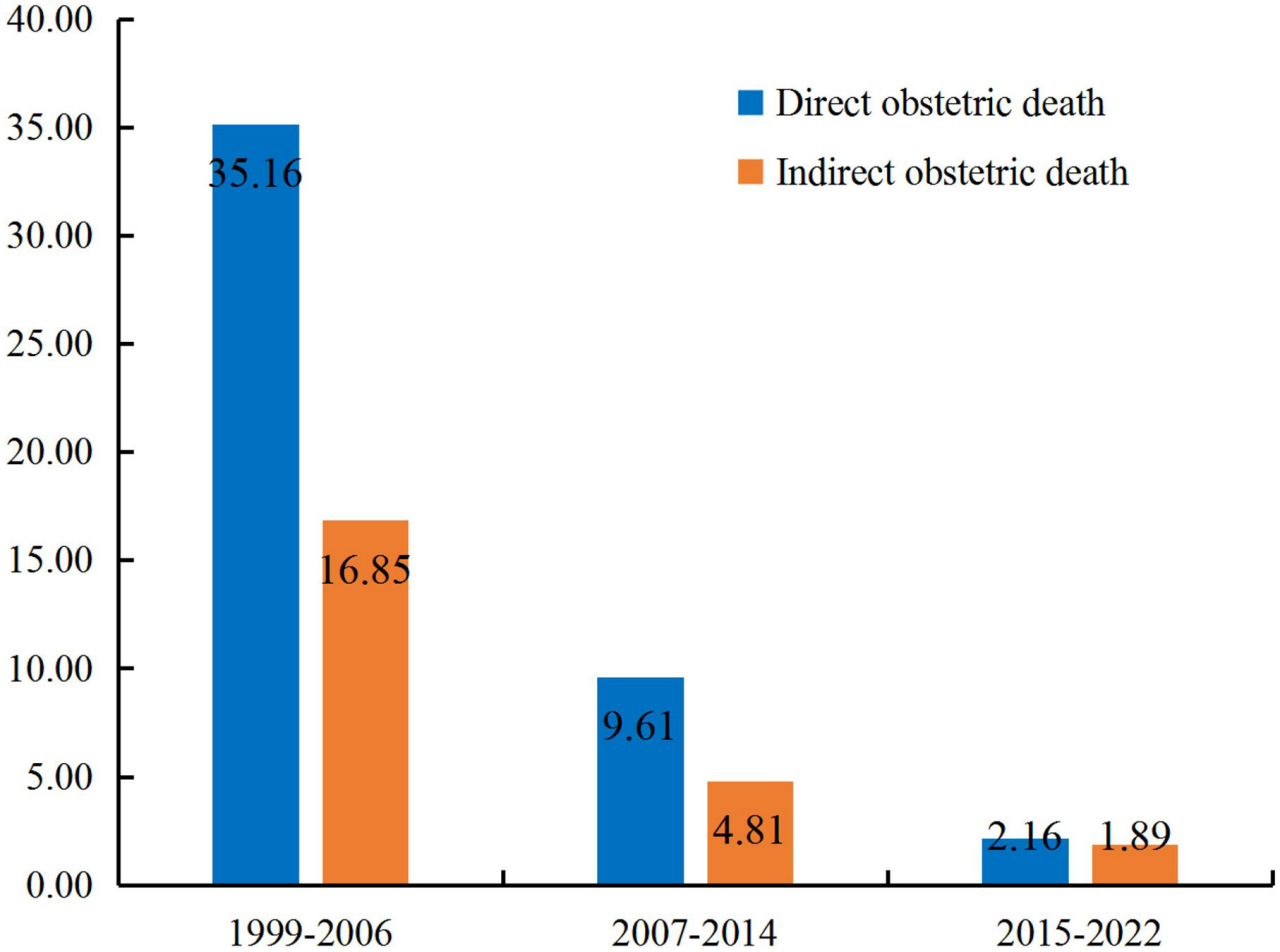
Differences of the MMR due to direct and indirect obstetric causes in different periods.

During 1999–2006, the MMR due to direct obstetric causes (35.16 per 100,000 live births) was 108.66% higher than that due to indirect obstetric causes (16.85 per 100,000 live births) (*χ^2^_trend_* = 8.805, *P* = 0.003), and the gap narrowed to 99.79% during 2007–2014. However, from 2015 to 2022, the MMR due to direct obstetric causes (2.16 per 100,000 live births, 95% CI 0.66–3.65) was only 14.29% higher than that due to indirect obstetric causes (1.89 per 100,000 live births, 95% CI 0.49–3.29), and the difference was of no significance (*χ^2^_trend_* = 0.067, *P* = 0.796) (Figure 3).

### 3.4. Trends of the MMR in major causes of maternal deaths

The four leading causes of maternal deaths during 1999–2022 included obstetric hemorrhage (4.41 per 100,000 live births), amniotic fluid embolism (3.37 per 100,000 live births), medical complications (2.44 per 100,000 live births) and pregnancy-induced hypertension (1.97 per 100,000 live births). When compared the MMR of the deaths causes in different period, the MMR due to all four major causes all shown statistical decreasing trends (*P* < 0.01) (Table 3), among which amniotic fluid embolism declined the most, with 84.33% decrease in 2015–2022 compared with in 1999–2006, while the MMR due to pregnancy-induced hypertension declined the least (59.55%), and it had replaced obstetric hemorrhage as the leading cause of deaths between 2015 and 2022.

**Table 3:** The MMR in major causes of maternal deaths in Baoan district during different period.

**Period**	**Obstetric hemorrhage**		**Amniotic fluid embolism**		**Medical complications**		**Pregnancy–induced hypertension**	
	**Maternal deaths (n)**	**MMR(95% CI)**	**Maternal deaths (n)**	**MMR(95% CI)**	**Maternal deaths (n)**	**MMR(95% CI)**	**Maternal deaths (n)**	**MMR(95% CI)**
1999–2006	20	14.65 (8.23–21.07)	15	10.99 (5.43–16.55)	12	8.79 (3.82–13.76)	9	6.59 (2.29–10.90)
2007–2014	15	4.24 (2.09–6.39)	13	3.68 (1.68–5.67)	8	2.26 (0.69–3.83)	4	1.13 (0.02–2.24)
2015–2022	3	0.81 (0.11–17.25)	1	0.27 (0.26–7.98)	1	0.27 (0.26–7.98)	4	1.08 (0.02–2.14)
Total	38	4.41 (3.01–5.82)	29	3.37 (2.14–4.59)	21	2.44 (1.40–3.48)	17	1.97 (1.04–2.91)
*χ^2^_trend_*		38.183		32.077		25.823		10.580
*P*		<0.01		<0.01		<0.01		<0.01

### 3.5. Trends of the constituent ratio in out-of-hospital delivery

There were 90 pregnant women had delivered before the death occurred, including 39 (43.33%) delivered out-of-hospital, and 51 (56.67%) delivered in hospital. Worth of note, none of the dead women delivered outside the hospital since 2015. The constituent ratio of out-of-hospital delivery significantly decreased by 100.00% in 2015–2022 when compared with in 1999–2006 (χ_*trend*_ = 8.832, *P* = 0.003) (Table 4).

**Table 4:** The constituent ratio differences of maternal deaths in Baoan district during different periods [n, (%)].

**Factors**	**1999–2006 (*N* = 71)**	**2007–2014 (*N* = 51)**	**2015–2022 (*N* = 15)**	**Total (*N* = 137)**
^#^ **Site of delivery**				
^*^Out–of–hospital	24 (55.81)	15 (39.47)	0 (0.00)	39 (43.33)
In–hospital	19 (44.19)	23 (60.53)	9 (100.00)	51 (56.67)
**Maternal age**				
<35	62 (87.32)	32 (62.75)	12 (80.00)	106 (77.37)
≥35	9 (12.68)	19 (37.25)	3 (20.00)	31 (22.63)
^†^ **Results from the review committee**				
Preventable death	61 (98.39)	41 (80.39)	9 (60.00)	90 (65.69)
Unpreventable death	1 (1.61)	10 (19.61)	6 (40.00)	47 (34.31)

^#^Only data of 90 maternal deaths were included because there were 90 had delivered among 137 deaths.

^*^Including at home, in illegal clinics and on the way to hospital.

^†^Only results between 2000 and 2022 were included because the maternal death review committee was established from 2000.

### 3.6. Trends of the constituent ratio in advanced maternal age

There were 31 (22.63%) maternal deaths with advanced age (≥35 years old) during 1999–2022, differences in the constituent ratio of advanced age in different periods were statistical significant (*χ^2^_trend_* = 3.999, *P* = 0.046), with 57.73% increased in 2015–2022 (20.00%) when compared to 1999–2006 (12.68%) (Table 4).

### 3.7. Trends of the constituent ratio in preventable death

A number of 128 deaths between 2000 and 2022 were assessed by the maternal mortality review committee. Among these, 111 (86.72%) were determined to be preventable deaths. the constituent ratio of preventable death significantly dropped by 39.02% in 2015–2022 (60.00%) compared with in 1999–2006 (98.39%) (*χ^2^_trend_* = 18.225, *P* < 0.001) (Table 4).

### 3.8. Trends of relevant indicators that affected the maternal deaths

In Baoan, the in-hospital delivery rate had raised from 99.11% in 2003 to 99.95% in 2022 (*χ^2^_trend_* = 1,774.327, *P* < 0.001, Supplementary Figure 1). The percentage of pregnancies being systematic managed increased from 21.11% in 2003 to 96.56% in 2022 (*χ^2^_trend_* = 220,112.813, *P* < 0.001, Supplementary Figure 2). Meanwhile, the proportion of pregnant women with advanced age had risen to 20.66% in 2022 compared to 4.26% in 2003 (*χ^2^_trend_* = 12,934.814, *P* < 0.001, Supplementary Figure 3).

## 4. Discussion

Our study shown that the MMR in Baoan district declined by 89.31% from 95.31 per 100,000 live births in 1999 to 10.19 per 100,000 live births in 2022, which had in advance met the Sustainable Development Goals (SDGs) target of pushing MMR down to 70 per 100,000 live births before 2030 (21). The annual decline rate of MMR (9.26%) in Baoan was much faster than the target pace in MDG5 Goal of 5.5% (21). Since 2010, the MMR in Baoan was lower than the 16 per 100,000 live births reported by the WHO for the average MMR in developed countries as a group (22). We also found that the constituent ratio in both preventable death and deaths among out-of-hospital delivery in Baoan shown a downward trend, the above results indicated that Baoan district had made encouraging progress in reducing maternal deaths in the past two decades.

The MMR in Baoan remained high before 2010 largely due to the deaths in migrant population during that period. As previously described (17, 23), the low economic status, weak health awareness /educational background of migrant population had made it challenging to improve maternal healthcare level. Although there was still a gap in MMR between permanent and migrant population in Baoan district, the gap between them had narrowed rapidly, and the decreased rate of MMR in migrant population (68.15%) was higher than that in permanent population (48.73%) during 1999–2022. This might be contributed by the continuous implementations of “Reducing Maternal Mortality and Eliminating Neonatal Tetanus” project and the in-hospital delivery subsidy project. Baoan had established a green channel for impoverished pregnant women to ensure certain fee waiver in prenatal examinations, in-hospital delivery and critical care since 2005, which had greatly reduced the economic burden of migrant population and promoted the in-hospital delivery rate in Baoan. Besides, the special actions which aimed to crackdown on illegal medical practices and illegal deliveries also played an important role in lowering the risk of maternal death during out-of-hospital delivery. After 2015, no maternal death caused by out-of-hospital delivery in Baoan. The findings of our study were in accordance with previous studies (24, 25) that in-hospital delivery was effective to prevent maternal deaths. The rapid reductions of MMR in Baoan were also attributed to the substantial efforts that all MCH institutions devoted to providing systematic and high-quality prenatal, delivery, and postpartum care, leading a significant upward trend in the percentage of pregnancies being systematic managed between 1999 and 2022.

Regardless of the maternal mortality being effectively controlled, with the process of population transform driven by industrial transform in Baoan, the changes of fertility concept (16), and the adjustment of national fertility policy (26, 27), further protecting maternal safety in Baoan faced many new challenges, and much work remains to be done in the coming decade.

Firstly, our study revealed that the gap of MMR due to direct and indirect obstetric causes had been narrowed to 14.29% during 2015–2022. This trend was consistent with the changes of causes of maternal deaths. More specifically, direct obstetric factors such as postpartum hemorrhage, amniotic fluid embolism or ectopic pregnancy had been controlled through years of efforts, but indirect obstetric factors that were more difficult for obstetricians to identify and deal with were gradually emerging (28). For instance, China had continuously adjusted and loosen the national COVID-19 prevention and control measures since December 7, 2022, which would pose new challenges to maternal mortality control. Of note, the first maternal death caused by COVID-19 in Baoan had occurred on December 19, 2022. Therefore, there is an urgent need for forming a multidisciplinary emergency team with well educated and highly trained obstetricians, midwives, respiratory doctors and experts from other relevant fields to deal with critical and severe pregnant women in time.

Secondly, we found that although obstetric hemorrhage remained the leading cause of maternal deaths, the MMR due to obstetric hemorrhage decreased by 76.50% in 2015–2022 when made a comparison to 1999–2006. The MMR due to amniotic fluid embolism, medical complications and pregnancy-induced hypertension also declined to varying degrees, and pregnancy-induced hypertension had replaced obstetric hemorrhage as the leading cause of deaths in recent years. All these had indicated that certain changes had occurred in the risk factors of maternal death during the social-demographic transition period from 1999 to 2022. To adjust to these changes, it is suggested to strengthen the training of obstetricians to improve the capacity of prediction, identification, diagnosis and treatment of obstetric diseases such as pregnancy-induced hypertension.

Thirdly, we found that the constituent ratio of maternal deaths with advanced age significantly increased by 57.78% in 2015–2022 compared with in 1999–2006. In order to actively respond to the aging population, to improve the population structure and to maintain the human resources advantages, China had implemented the “universal two-child” policy on January 1, 2016 (26), and further adjusted it to “universal three-child” policy (27) on May 31, 2021. Women of reproductive age who had already given birth to one or two children were targeted by the policies, which would have stimulated births to older mothers. As is generally known, age is considered to be one of the important factors affecting maternal mortality (29). Many studies pointed out that pregnant women over 35 years old had a higher risk for many pregnancy complications and poor birth outcomes (29, 30). In Baoan, the proportion of pregnant women with advanced age had risen by 66.88% from 12.38% in 2016 to 20.66% in 2022, which might possibly lead to a rebound in maternal mortality. Therefore, it is essential to strengthen the prenatal consultation, evaluation and high-risk screening, improve the treatment and referral capacity for critical and severe cases. It is also necessary to further improve the elder pregnant women's health awareness through in-depth health education project.

There were several limitations of this analysis that need to be mentioned. First, due to the wide year span and the imperfect data-collection mechanism in the early years, information on maternal deaths was not collected in a consistent form, resulting in some missing indicators that were of great analytical value. Second, descriptions about the demographic characteristics of the maternal deaths were limited, largely due to the natural limitations of SZMCHS (17), routine data collections in SZMCHS were primarily used for administrative or governmental reports rather than research. For instance, socioeconomic indicators such as income level and occupation were often missing or inaccurate in SZMCHS due to the lack of accurate measurement or definition. Last but not the least, considering the stability of statistical efficiency, we were unable to conduct further grouping for analysis with the limited sample sizes of maternal deaths in our report. The aforementioned flaws all highlighted the importance and necessity of enhanced data collection and an updated system for recording detailed information for every death case.

## 5. Conclusion

Baoan district had made encouraging progress in improving maternal survival, especially in migrant population. To further reduce the MMR under the new challenges in the coming era, strengthening professional training to improve the capacity of obstetricians and physicians, increasing the awareness and ability of self-help health care among elderly pregnant women were in urgent need for protecting motherhood safety.

## Data availability statement

The original contributions presented in the study are included in the article/Supplementary material, further inquiries can be directed to the corresponding author.

## Ethics statement

This study was reviewed and approved by the Ethics Committee of Baoan Women's and Children's Hospital (No. LLSC-2023-01-02-03-KS). The data used in this study was anonymous before its use.

## Author contributions

YL: conceived the study and provided overall guidance. WW and YZ: conducted the study, collected the data, and wrote the manuscript. YC: did the analysis. XC: reviewed each section in detail. All authors contributed to the article and approved the submitted version.
