# Supplementary figures and images for: Levels and trends of maternal death in Baoan district, Shenzhen, China, 1999–2022
Source: Front Public Health. 2023 Apr 17;11:1051717. doi: 10.3389/fpubh.2023.1051717 (PMC10149838; doi:10.3389/fpubh.2023.1051717)

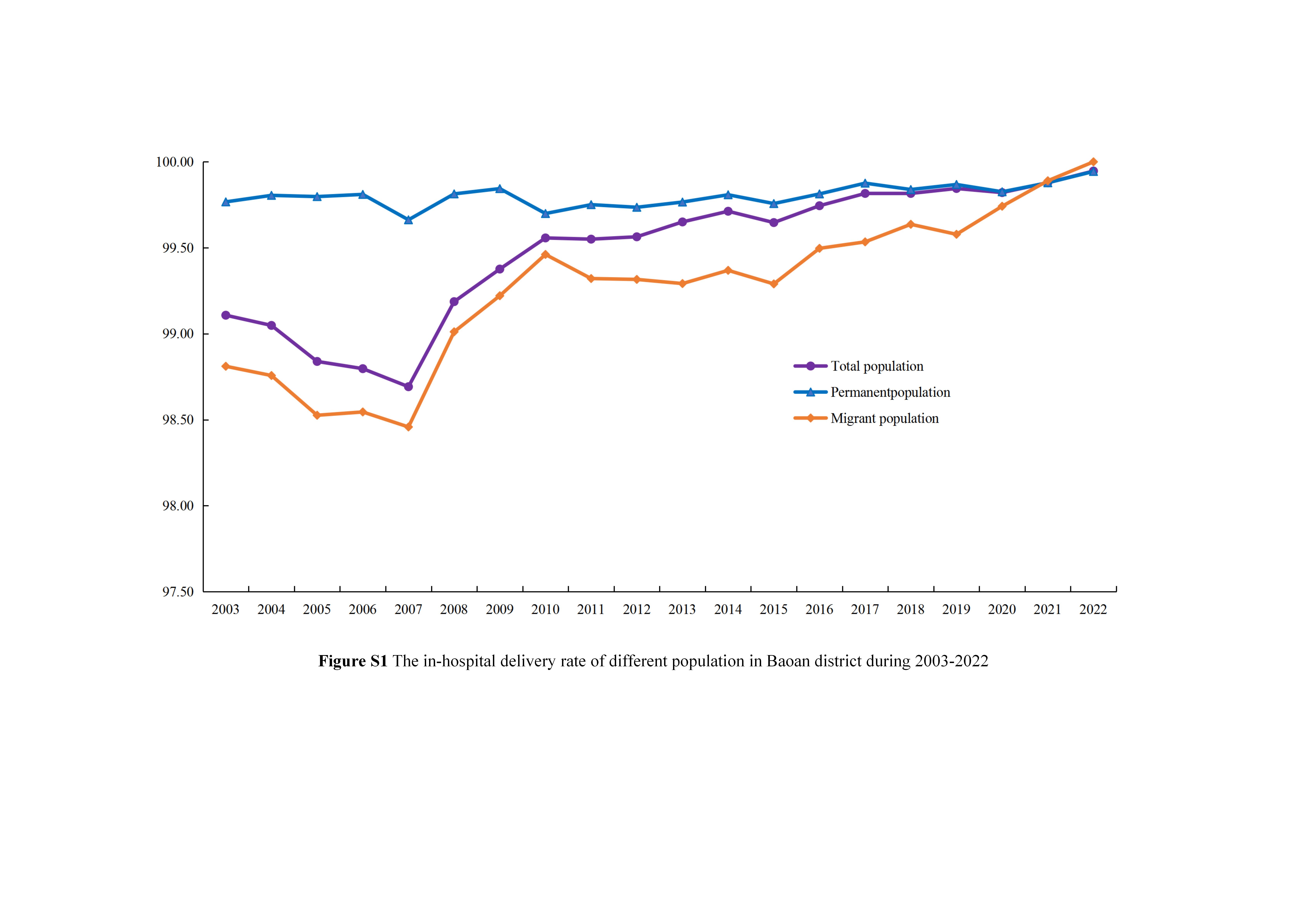

Supplement: Supplementary file 1 [file Image_1.JPEG]

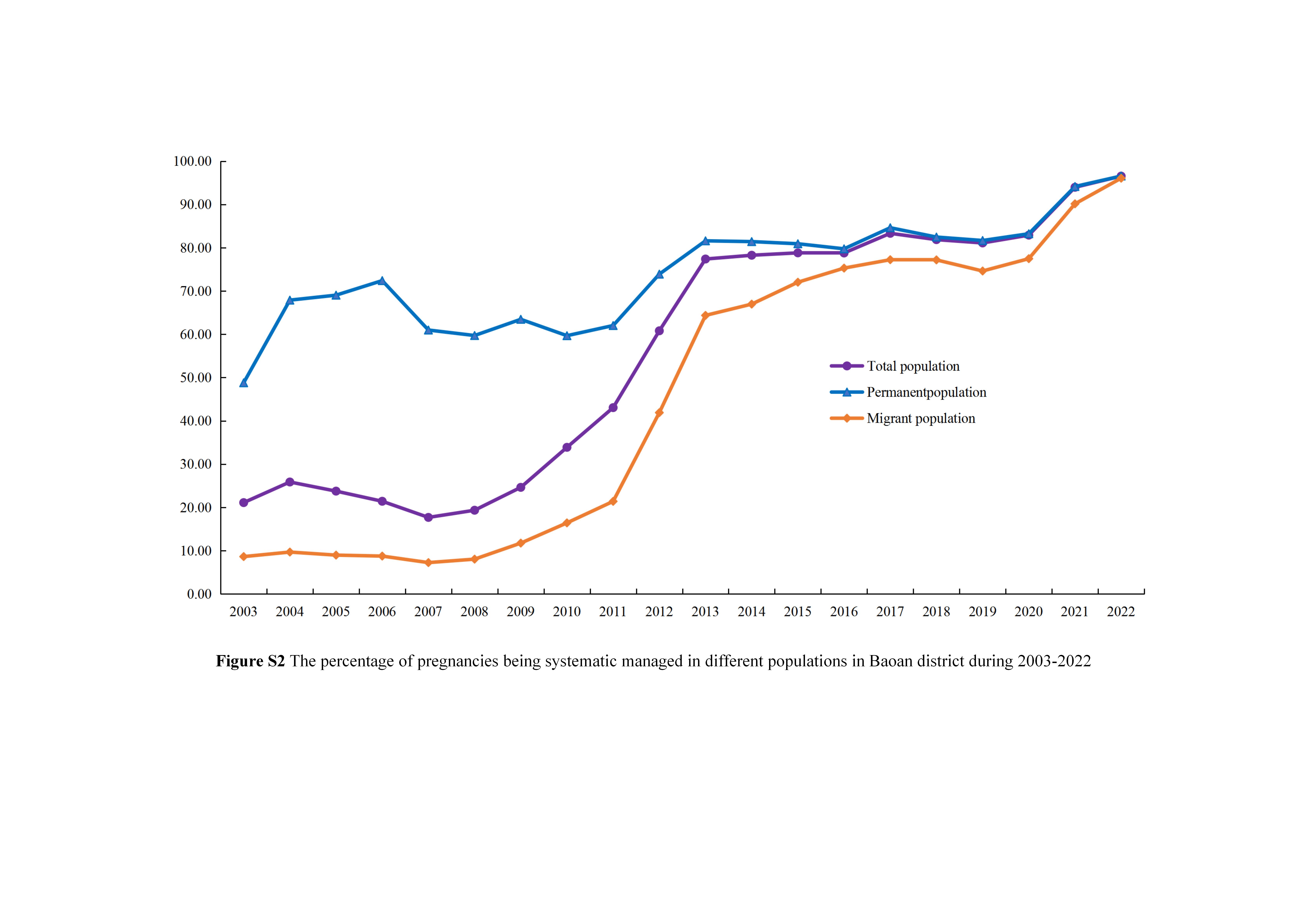

Supplement: Supplementary file 2 [file Image_2.JPEG]

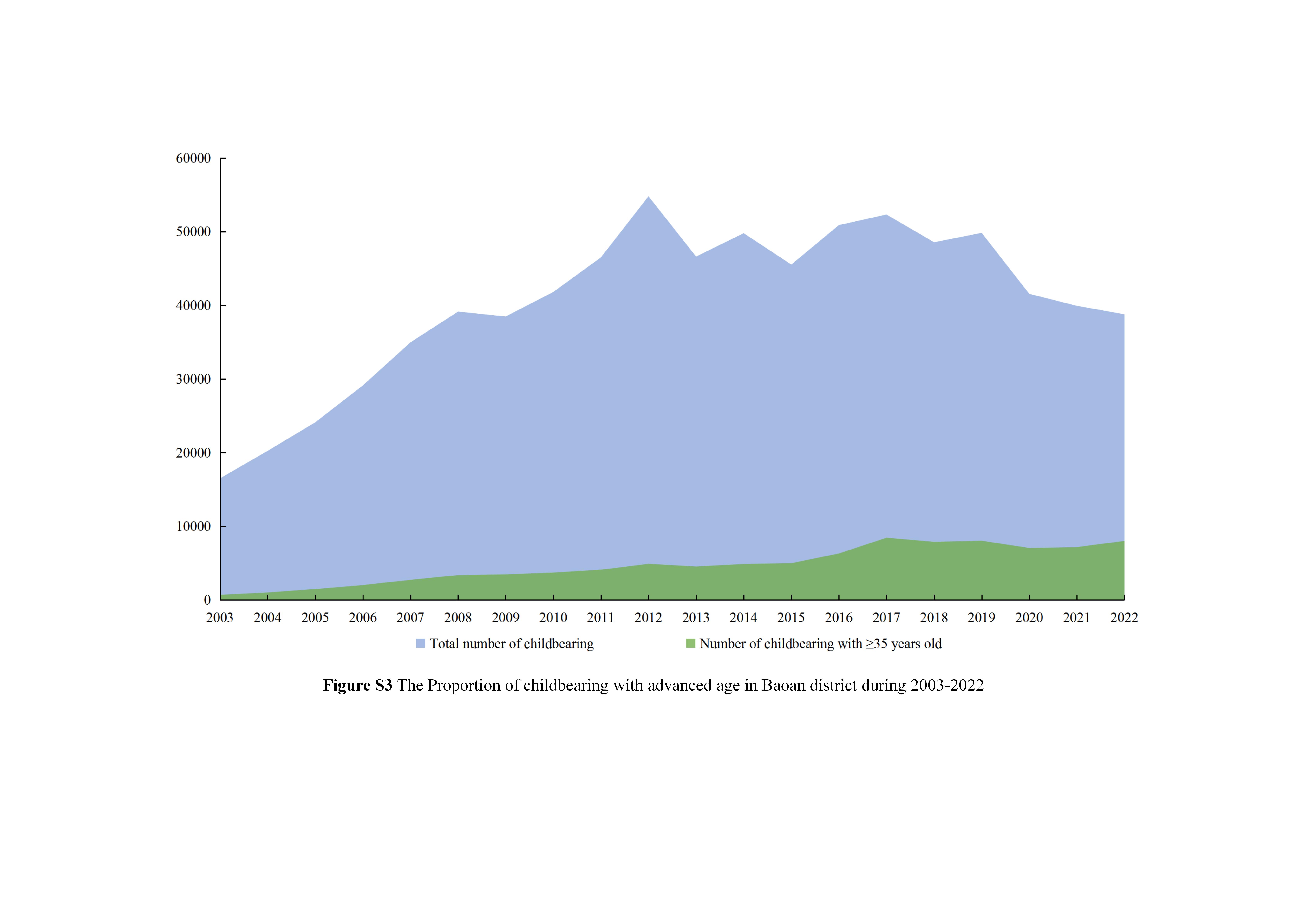

Supplement: Supplementary file 3 [file Image_3.JPEG]
